# Supplementary material for: Multisystemic Cellular Tropism of SARS-CoV-2 in Autopsies of COVID-19 Patients
Source: Cells. 2021 Jul 27;10(8):1900. doi: 10.3390/cells10081900 (PMC8394956; doi:10.3390/cells10081900)
Supplement: Supplementary file 1 [file cells-10-01900-s001.zip › cells-1287027 supplementary proofreading back 5.pdf]

Multisystemic Cellular Tropism of SARS-CoV-2 in Autopsies of COVID-19 Patients

Dickson W. L. Wong [1,†], Barbara M. Klinkhammer [1,†], Sonja Djudjaj [1,†], Sophia Villwock [1], M. Cherelle Timm [1], Eva M. Buhl [1,2], Sophie Wucherpfennig [1], Claudio Cacchi [1], Till Braunschweig [1], Ruth Knüchel-Clarke [1], Danny Jonigk [3,4], Christopher Werlein [4], Roman D. Bülow [1], Edgar Dahl [1], Saskia von Stillfried [1,*,†] and Peter Boor [1,2,5,*,†]

1 Institute of Pathology, RWTH Aachen University Hospital, 52074 Aachen, Germany; dwong@ukaachen.de (D.W.L.W.); bklinkhammer@ukaachen.de (B.M.K.); sdjudjaj@ukaachen.de (S.D.); svillwock@ukaachen.de (S.V.); mtimm@ukaachen.de (M.C.T.); ebuhl@ukaachen.de (E.M.B.); swucherpfenn@ukaachen.de (S.W.); ccacchi@ukaachen.de (C.C.); tbraunschweig@ukaachen.de (T.B.); rknuechel-clarke@ukaachen.de (R.K.-C.); rbuelow@ukaachen.de (R.D.B.); edahl@ukaachen.de (E.D.)
2 Electron Microscopy Facility, RWTH Aachen University Hospital, 52074 Aachen, Germany
3 Institute of Pathology, Hannover Medical School, 30625 Hannover, Germany; jonigk.danny@mh-hannover.de (D.J.); Werlein.Christopher@mh-hannover.de (C.W.)
4 Member of the German Center for Lung Research (DZL), Biomedical Research in Endstage and Obstructive Lung Disease Hannover (BREATH), 30625 Hannover, Germany
5 Department of Nephrology and Immunology, RWTH Aachen University Hospital, 52074 Aachen, Germany
* Correspondence: svonstillfri@ukaachen.de (S.v.S.); pboor@ukaachen.de (P.B.); Tel: +49-241-80-85227 (P.B.) ; Fax: +49-241-80-82446 (P.B.)
† Equally contributed

Supplementary information

Supplementary Method

External study cohort

Table S1. List of autopsies from different decedents organized in the TMA.

Table S2. Major pathological findings in the autopsy cohort.

Table S3. Patient characteristics of the external COVID-19 study cohort.

Figure S1. Validation of FISH detection on human autopsies.

Figure S2. Virus detection in respiratory system by FISH.

Figure S3. Virus detection in respiratory system by IHC staining.

Figure S4. Validation of CISH detection on external human autopsies.

Figure S5. Detection of SARS-CoV-2 sense, antisense, ACE2 and TMPRSS2 on an external sample by CISH.

Supplementary Method

SARS-CoV-2 RNA detection with Chromogenic in situ hybridization (CISH)

We performed CISH staining on the 1-µm-thick paraffin tissue microarray sections (n = 9 patients) received from an external COVID-19 autopsy center (provided by Prof. D. Jonigk, Institute of Pathology, Hannover Medical School) with the RNAscope® 2.5 HD Duplex Reagent Kit (Advanced Cell Diagnostics, Inc., Hayward, California, USA). The procedure is analogous to FISH until the amplifier steps. Instead of fluorophore, horseradish peroxidase (HRP) and alkaline phosphatase (AP) were conjugated to the C1 (cyan)

and C2 (red) probe, respectively. We applied the same probes used in FISH staining to the tissues. Lastly, tissue slides were counterstained with Gills Haematoxylin (Sigma-Aldrich, St. Louis, MO, USA) and mounted with EcoMount solution (Biocare Medical; Concord, California, USA). Stained slides were analyzed with Aperio VERSA Brightfield, Fluorescence & FISH Digital Pathology Scanner (Leica Microsystems Inc, Buffalo Grove, IL, USA).

External study cohort

The main characteristics of the external study cohort (9 male/ 4 female; median age 76 (54–96) years) are given in Table S3. The period between the hospital admission and death ranged from 3 to 22 days (median 9 days, Figure 1). 12 patients had a previous diagnosis of hypertension (n=11) and/ or diabetes mellitus type 2 (n=5). Four patients had a history of tobacco abuse, four patients were non-smokers, while no information on current or previous tobacco abuse was available for the remaining five patients. Six patients were treated with mechanical ventilation. One patient refused mechanical ventilation. Five patients received no ventilation therapy due to a palliative situation.

Table S1. List of autopsies from different decedents organized in the TMA blocks. Autopsies were processed with RT-PCR (P) and FISH staining (F). Tissues that were not available are labelled as (N/A).

**Patient/ Sex**	**1/ M**	**2/ F**	**3/ M**	**4/ M**	**5/ M**	**6/ F**	**7/ M**	**8/ F**
Lung central	P, F	P, F	P, F	P, F	P, F	P, F	P, F	P, F
Lung periphery	P, F	P, F	P, F	P, F	P, F	P, F	P, F	P, F
Distal trachea	P, F	P, F	P, F	P, F	P, F	P, F	P, F	P, F
Heart	P, F	P, F	P, F	P, F	P, F	P, F	P, F	P, F
Kidney	P, F	P, F	P, F	P, F	P, F	P, F	F	P, F
Liver	P, F	P, F	P, F	P, F	P, F	P, F	P, F	P, F
Adrenal gland	P, F	P, F	P, F	P, F	P, F	P, F	P, F	P, F
Spleen	P, F	P, F	P, F	P, F	P, F	P, F	P, F	P, F
Small bowel	F	F	N/A	F	F	F	F	F
Large bowel	F	F	F	P, F	F	F	F	F
Lymph node (perihilar)	P, F	P, F	P, F	P, F	P, F	P, F	P, F	F
Salivary gland	P, F	P, F	P, F	P, F	P, F	P, F	P, F	P, F
Tonsil	P, F	P, F	P, F	P, F	P, F	P, F	P, F	P
Bone Marrow	P	P	N/A	P	P, F	N/A	N/A	N/A
Ovary	N/A	P, F	N/A	N/A	N/A	N/A	N/A	F
Testicles	P, F	N/A	P, F	P, F	F	N/A	P, F	P, F
Uterus	N/A	F	N/A	N/A	N/A	P, F	N/A	P, F
Prostate	P, F	N/A	P, F	P, F	F	N/A	F	N/A
Urinary bladder	N/A	N/A	N/A	N/A	F	N/A	N/A	N/A
Oropharynx	P, F	P, F	F	P, F	P, F	P, F	P, F	P, F
Esophagus	P, F	P, F	P	P, F	P, F	P, F	P, F	P, F
Stomach	P, F	P, F	P, F	P, F	P, F	P, F	P, F	P, F
Pancreas	P, F	P, F	P, F	P, F	P, F	P, F	P, F	P, F
Thyroid	P, F	P, F	P, F	P, F	P, F	P, F	P, F	P, F
Muscle	P, F	F	P, F	P, F	F	P, F	P, F	P
Skin	P	F	P, F	P, F	N/A	P, F	P, F	P, F
Lymph node (mesenteric)	P, F	P, F	P, F	P, F	P, F	P, F	N/A	P

Table S2. Major pathological findings in the autopsy cohort.

**Pulmonary**							**Extra-pulmonary**		
							Cardiovascular	Urogenital	Gastrointestinal
							Heart	Kidney	Liver
Patient/ sex	DAD	Inflammation	Giant cells	Squamous metaplasia	Hyaline membranes	Additional observation			
1/ M	Proliferative/ organizing phase	Interstitial infiltrate	+	+	+	Intraalveolar edema, focal organized pneumonia	Normal heart weight, fibrosis in endocardium, patchy myocardium infiltrate	Type 1 diabetes and nodular glomerulosclerosis, no significant chronic inflammation	Steatosis, intracytoplasmic cholestasis
2/ F	Proliferative/ organizing phase	Interstitial lymphocytic inflammation	-	++	+	Hyaline microthrombi,	Slight cardiomegaly, patchy lymphocytic infiltrate in epicardium, macrophages (signs of previous bleeding), absorptive/ reactive inflammation, endocardial fibrosis, discrete pericarditis, perivascular fibrosis, signs of cellular hypertrophy	Inflammatory cells only in fibrotic areas	Liver cirrhosis, chronic inflammation in capsule and fibrotic areas, steatosis
3/ M	Proliferative phase	Superinfection with acute inflammation	-	-	+ (very focal)	Intra-alveolar edema, Intra-alveolar hemorrhage	Cardiomegaly, cellular hypertrophy, very scarce lymphocytes, single cell necrosis, focal fibrosis	Hypertensive damage, inflammation only in fibrotic areas, no peritubular capillaritis	Disseminated inflammation, steatosis
4/ M	Proliferative/ organizing phase	Interstitial inflammation	+	+	-	Hemorrhage, thromboembolus in small pulmonary arteries	Cardiomegaly, perivascular lymphocyte, eosinophilic neutrophil infiltrate, discrete chronic inflammation	Chronic inflammation, single sclerotic glomeruli, acute kidney injury	Cholangitis lenta, chronic inflammation of biliary ducts
5 / M	Proliferative/ organizing	Acute pneumonia with granulocytes, macrophage, acute inflammation	+	+	+	Intraalveolar edema, intra-alveolar hemorrhage	Cardiomegaly, patchy myocardial infiltrate, histiocytic infiltrate, discrete chronic inflammation	Chronic inflammation, single sclerotic glomeruli, acute kidney injury, peritubular capillaries/ vasa recta with lots of leukocytes	Cholangitis lenta, chronic inflammation of biliary ducts
6/ F	Proliferative phase	Interstitial inflammation	++	+	+	Thrombus, Lung metastasis, intraalveolar edema, bronchitis	Slight cardiomegaly, patchy myocardial infiltrate	Patchy inflammatory infiltrate, some peritubular capillaries with leukocytes	Lymphocytes in sinusoids, no other pathology
7/ M	Proliferative and fibrotic phase	Intra-alveolar granulocytes	++	+	-	Intraalveolar edema,	Normal heart weight, histiocytic infiltrate	Focal chronic inflammation, primarily in fibrotic areas,	Cholangitis lenta, chronic inflammation of biliary ducts

								some in peritubular capillaries	
8/ F	Proliferative and fibrotic phase	Granulocytes, chronic inflammatory infiltrate	-	-	+	Intraalveolar hemorrhage, intraalveolar edema	Slight cardiomegaly, inflammation only in fibrosis	Rejection, cirrhotic kidney	Perivascular and sinusoidal lymphocytes

Table S3. Patient characteristics of the external COVID-19 study cohort.

Patient	Sex	Age range (y)	BMI	Smoker	Hospital admission to death (d)	Ventilation therapy
1	M	91-95	Underweight	No	14	No
2	M	76-80	Moderately obese	n.a.	22	Yes
3	M	71-75	Severely obese	n.a.	12	Yes
4	F	66-70	n.a.	n.a.	14	n.a.
5	F	76-80	n.a.	n.a.	7	Yes
6	M	51-55	n.a.	n.a.	21	Yes
7	M	76-80	Severely obese	Yes	3	Yes
8	M	66-70	Overweight	Yes	9	Yes
9	M	71-75	Overweight	Yes	3	No
10	F	81-85	Overweight	Yes	4	No
11	F	66-70	Severely obese	No	9	No
12	M	86-90	Overweight	No	5	No
13	M	96-100	Normal weight	No	3	No

BMI = Body-mass index; d = day; y = year.

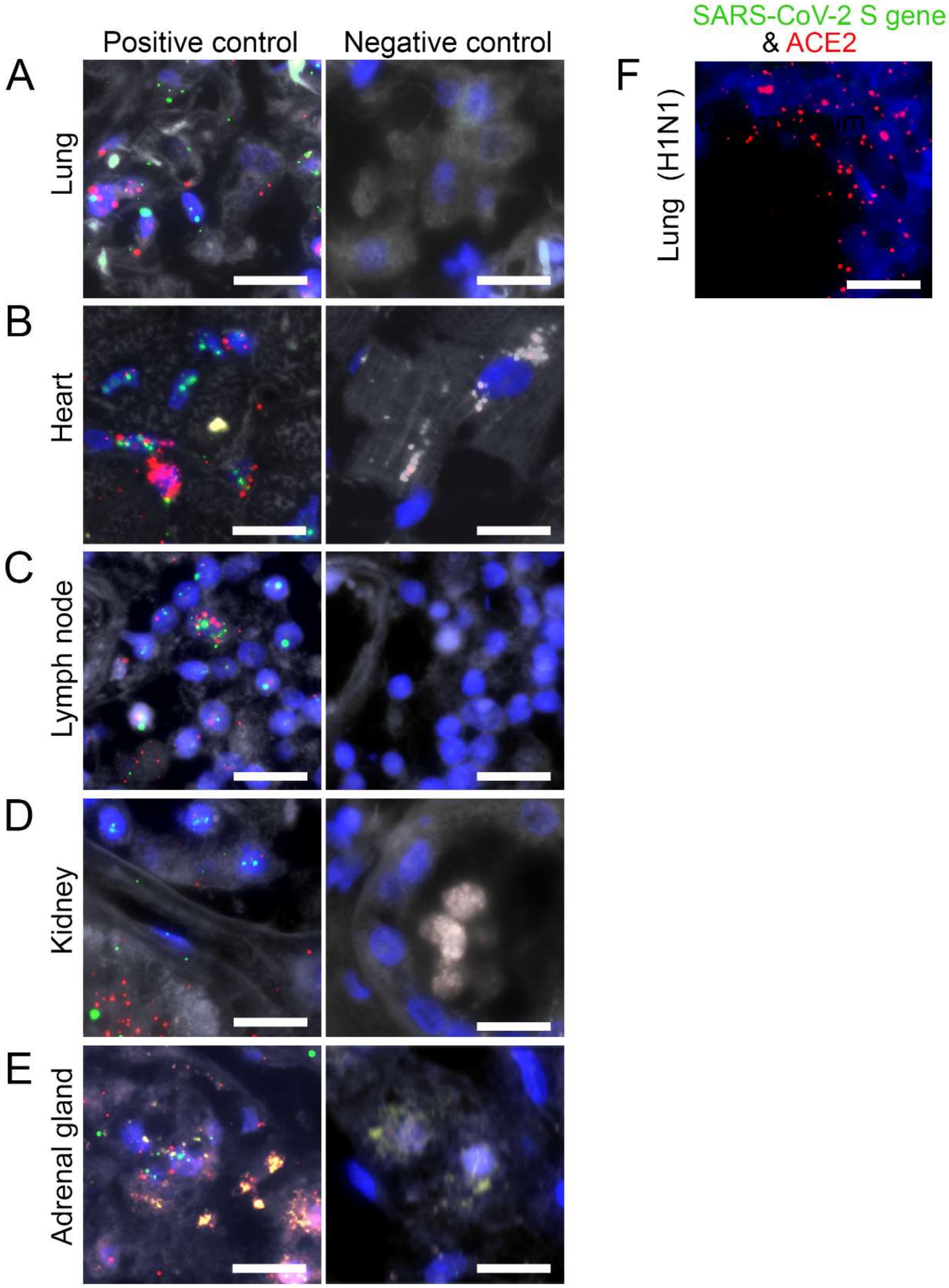

Figure S1. Validation of FISH detection on human autopsies. FISH method was validated by incubation of probes targeting *Homo sapiens POLR2A* (green) and *PPIB* (red) genes as positive controls, and *dap* gene of *Bacillus subtilis* as a negative control in the lung (A), heart (B), lymph node (C), kidney (D) and adrenal gland (E) autopsies. Probes against SARS-CoV-2 (green; absent) and *Homo sapiens ACE2* (red) were incubated with a lung autopsy collected from a patient diagnosed with influenza A virus subtype H1N1 before COVID-19 outbreak (F). Scale bars represent 20 μm.

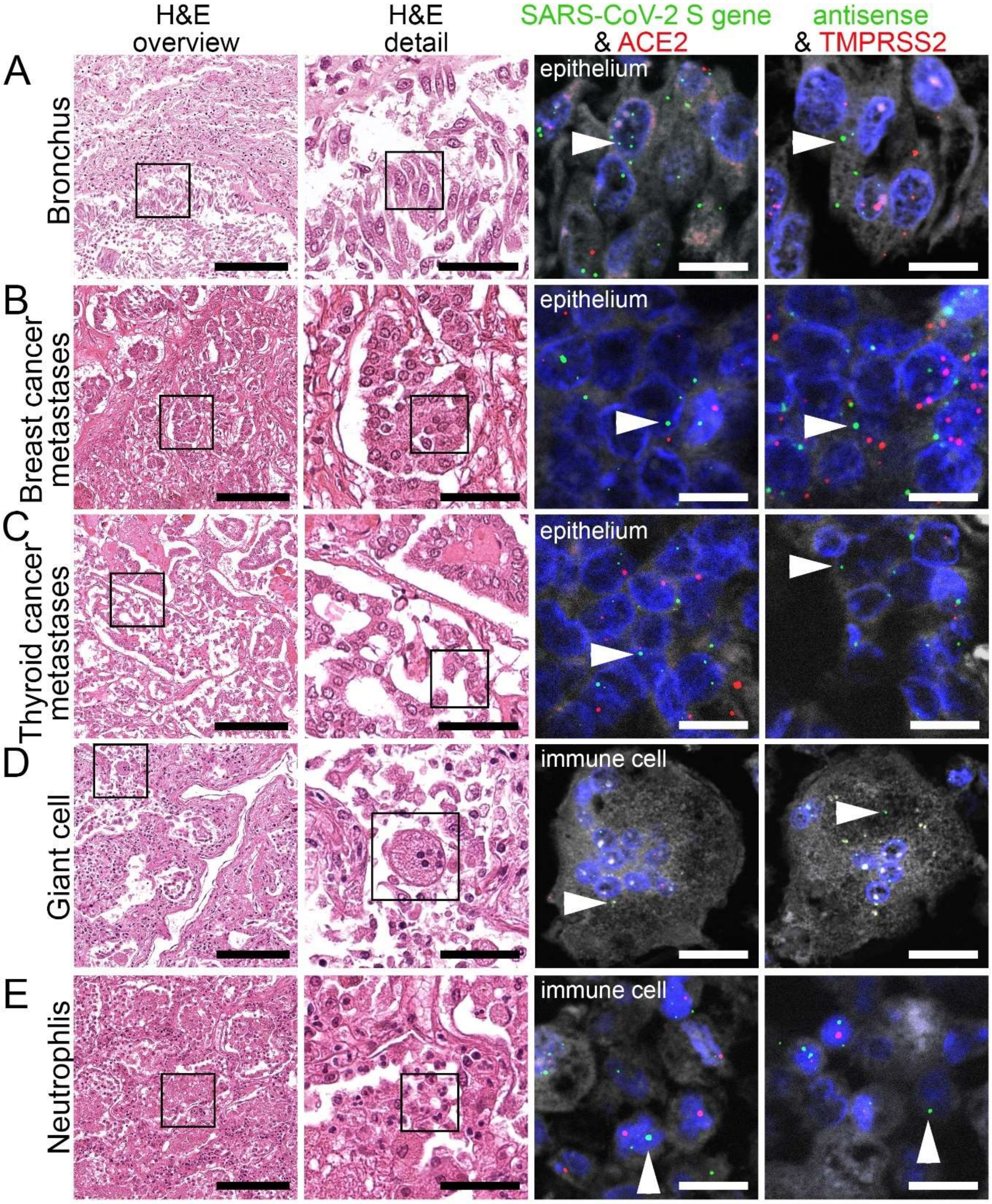

Figure S2. Virus detection in respiratory system by FISH. HE stained lung tissue and representative image sections showing FISH co-visualization of RNA sequences either of SARS-CoV-2 S gene genomic RNA (green, arrowhead) and ACE2 (red) or SARS-CoV-2 antisense strand replicating RNA (green, arrowhead) and TMPRSS2 (red). Morphological details are shown in regions of a bronchus (A), metastasis from breast cancer (B) and thyroid cancer (C) and immune cells, i.e., multinucleated giant cells (D) and neutrophils (E). Scale bars represent 200, 50 and 10 μm (A, B, C, D, F) or 20 μm (E), respectively.

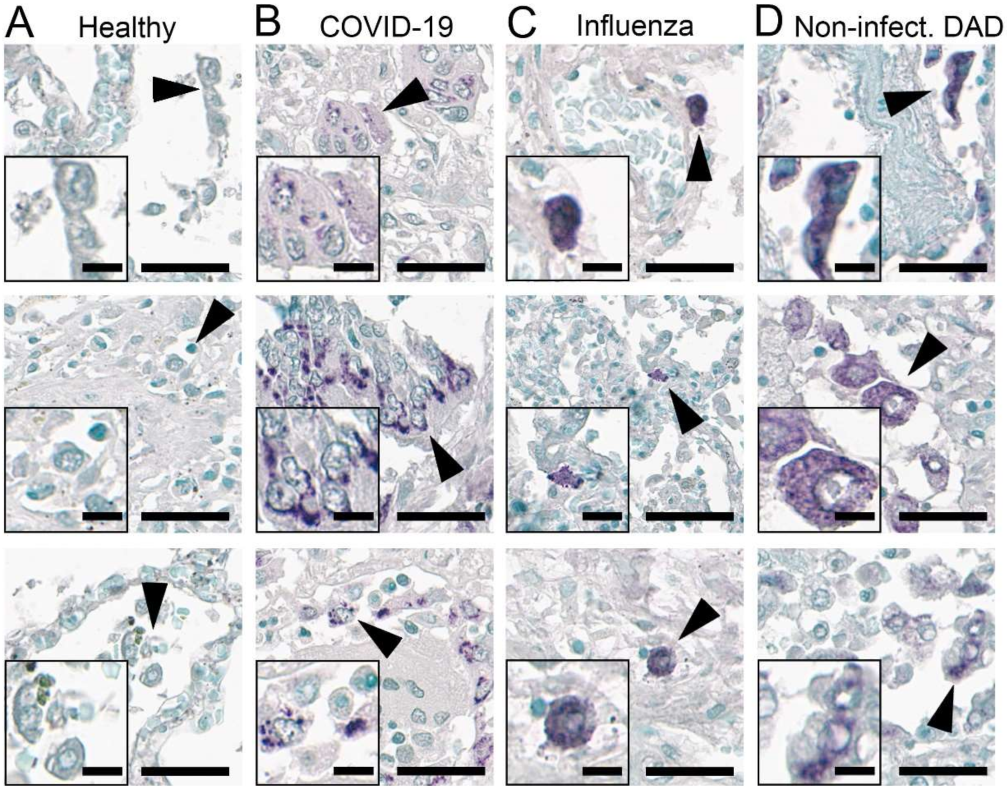

Figure S3. Virus detection in the respiratory system by IHC staining. Representative IHC staining of SARS spike glycoprotein (#Ab272420) in the autopsy lung tissues collected from different patients in each group of our cohort, consisting of patients without pulmonary disease/ healthy lung (A), COVID-19 patients (B), patients infected with influenza A virus subtype H1N1 (C) or diffuse alveolar damage (non-infectious, D). Scale bars represent 40 μm and 10 μm (insert).

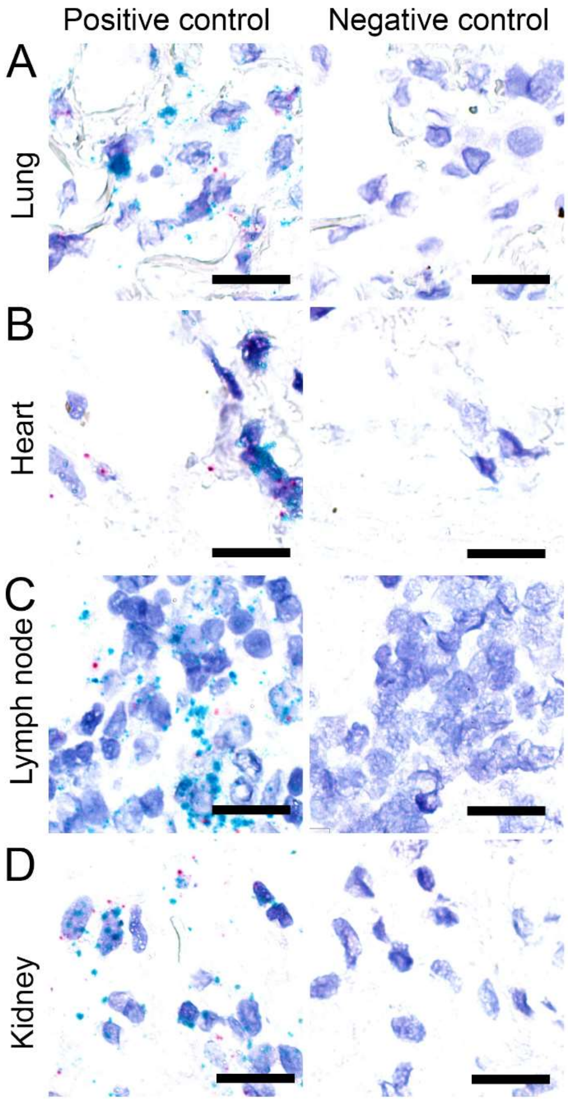

Figure S4. Validation of CISH detection on external human autopsies. CISH method was validated by incubation of probes targeting *Homo sapiens POLR2A* (cyan) and *PPIB* (red) genes as positive controls, and *dap* gene of *Bacillus subtilis* as a negative control in the lung (A), heart (B), lymph node (C) and kidney (D) autopsies. Scale bars represent 20 μm.

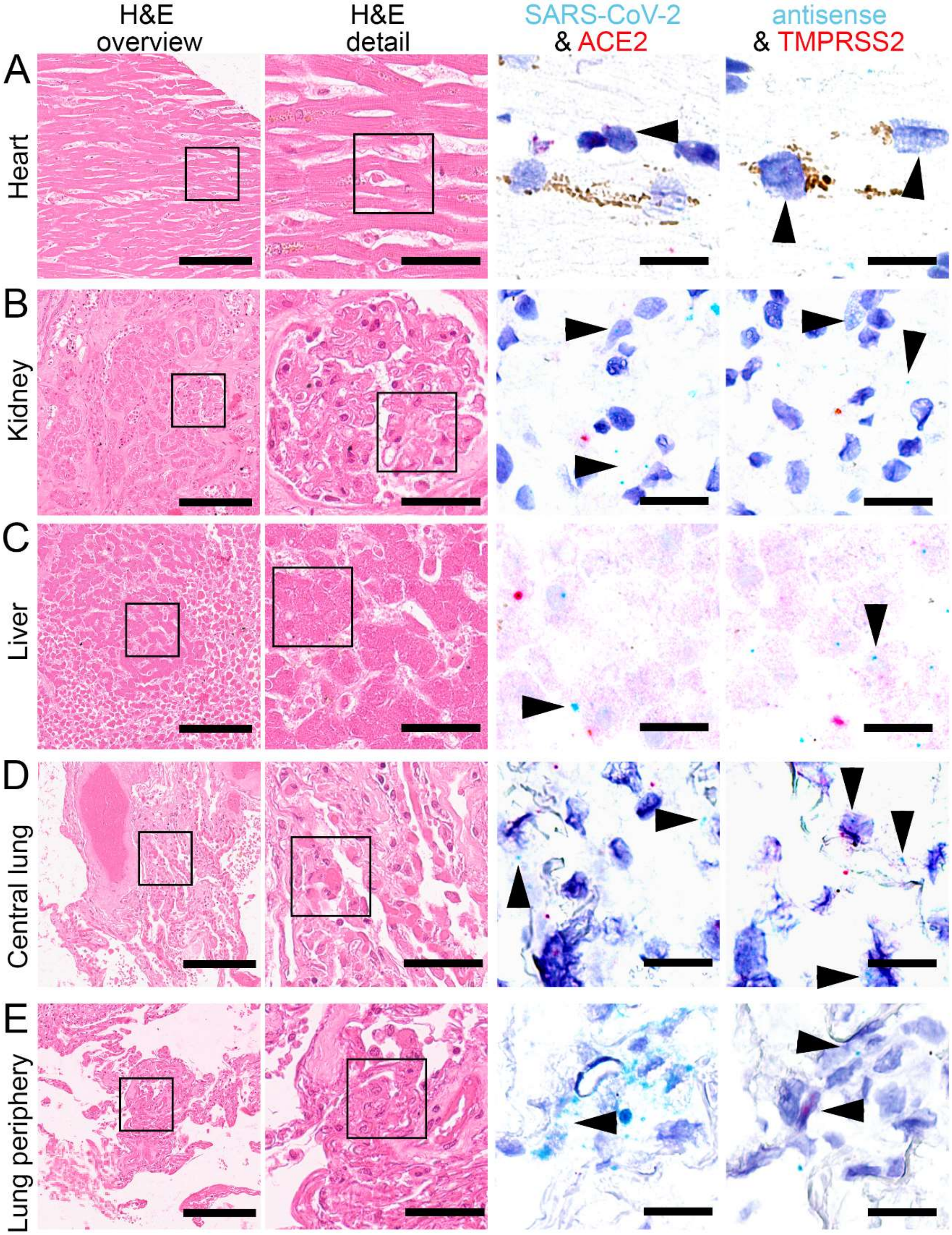
H&E overview
H&E detail
SARS-CoV-2 & ACE2
antisense & TMPRSS2
A
Heart
B
Kidney
C
Liver
D
Central lung
E
Lung periphery

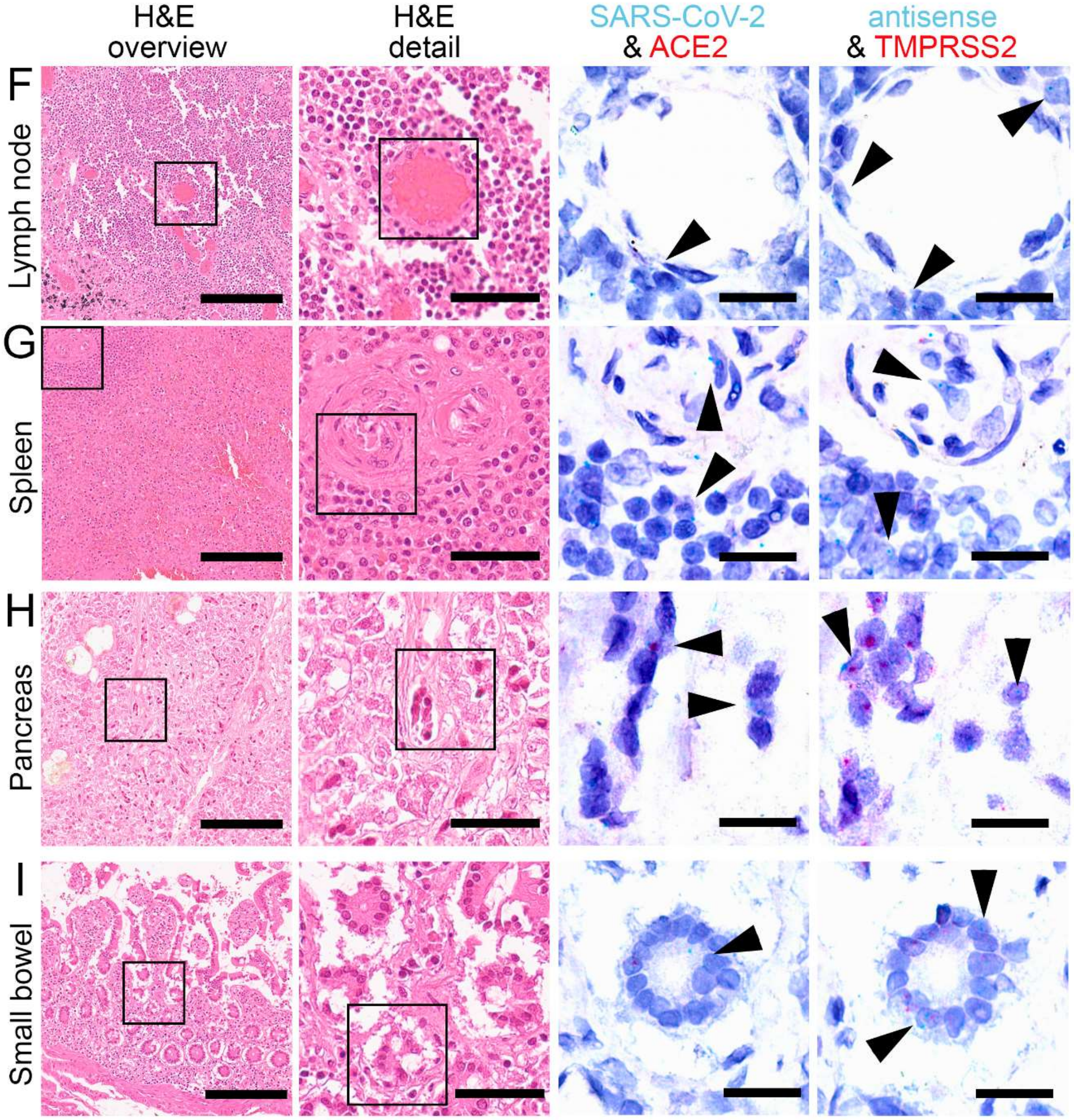

Figure S5. Detection of SARS-CoV-2 sense, antisense, ACE2 and TMPRSS2 on external samples by CISH. HE stained tissue and representative image sections showing CISH co-visualization of RNA sequences either of SARS-CoV-2 S gene genomic RNA (cyan, arrowhead) and ACE2 (red) or SARS-CoV-2 antisense strand RNA indicating replicating virus (cyan, arrowhead) and TMPRSS2 (red) in the heart (A, cardiomyocyte), kidney (B, glomerulus), liver (C, hepatocyte), central lung (D, alveolus), lung periphery (E, alveolus), lymph node (F, capillary endothelial cells), spleen (G, capillary endothelial cells), pancreas (H, ductal epithelial cells) and small bowel (I, enterocytes). Scale bars represent 200, 50 and 20 μm, respectively.
